# Letter from Ben C. Miller, M. D.

**Published:** 1876-08

**Authors:** Ben C. Miller

**Affiliations:** Sanitary Sup’t.


Editors of the Chicago Medical Journal and Examiner:

In your June number, in the report of Dr. Danforth on the Pathological Anatomy and Histology of Cholera, he speaks of "Dr. McClellan coolly ignoring the work of the Society of Physicians and Surgeons, and crediting the investigations to the Chicago Board Health."

As this places Dr. McClellan, as well as myself, in a false position, I desire to state the circumstances as they occurred.

During the epidemic of 1873 Dr. Simons was appointed Assistant Sanitary Inspector, with instructions to investigate fully the causes of the disease, and to make *post mortem* examination of all cases, when one could be obtained, as I desired to have an examination made of the specimens obtained. He was directed to do this as an employee of this Department, and not as a member of the committee. When the *post mortems* were made, he informed me that he had invited Dr. Hyde to be present, and he had conducted the examinations and had the specimens in his possession.

I then saw Dr. Danforth and requested him to make an examination, and report the same for publication in the report of the Board of Health. The Department paying all expenses connected with the examination, and retaining all the plates; and furnishing Dr. Danforth with a number of bound copies of his report for distribution.

When Dr. McClellan was here he spoke to Dr. Danforth in my presence, in regard to inserting his report, as published in the Board of Health Report, in the work on cholera; stating that he would give Dr. Danforth credit for the same, and that this would give a larger audience than publishing the same in the MEDICAL JOURNAL.* To this Dr. Danforth assented, and if he had desired, one word from him would have given the Society all the credit that has been given to Dr. Danforth. It is true the Board of Health did not order any *post mortems*; but, as the executive officer of the Board, I did; and, in inviting Dr. Hyde to be present, Dr. Simons was acting in his official capacity, and permitted Dr. Hyde to do what he had been ordered to do. I was gratified when I learned that Dr. Hyde was present and took charge of the examinations, but did not approve of Dr. Simons' permitting him to take the specimens away with him, for by this it would appear that his first duty was to the Society, and not to the Department that was paying him for this service.

In the report of the Board of Health, which contains the report of Dr. Danforth (pages 30 to 44), due credit was given to Dr. Danforth, also to Dr. Hyde for making the *post mortems*.

If Dr. Danforth had desired the Society to have the credit instead of himself, he had access to the proofs and corrected them, why did he not made the change then? I think it illy becomes him, at this late day, to arraign Dr. McClellan for not accrediting the Society of Physicians and Surgeon with the investigations, and stating that the Board of Health had not the slightest connection with the inauguration or prosecution of these researches, and that it was done wholly under

* Dr. McClellan writes me as follows: "Dr. Danforth stated to me that the notes had been carelessly written, that he was not satisfied with them, that he would go over the entire ground, rewrite the paper and send it *to me*. Athough I wrote him several times on the subject, and many times wrote you to see him about it, I never received one line from him or from anyone else."

the auspicies of the Society of Physicians and Surgeons; when the Health Department was in constant communication with him, and paid all bills connected with the examination, and now have possession of all the plates.

Further, it illy becomes him to bring Dr. McClellan into this, when he kept the report out of the JOURNAL, and permitted Dr. McClellan to take it, not as a report of the Society, but as his report, because it would give him the greater audience.

I thank Dr. Hyde for his examinations, for they were made thoroughly; although Dr. Simons had been ordered to do the same work; and if in the Health Report Dr. Hyde has been neglected in any way I am ready to make the amende honorable. But Dr. Danforth is rather late in raking up old scores. At that time the only objection he made was that I gave him the plain title of "Dr. I. N. Danforth," and did not append his title of "Lecturer in Rush Medical College."

In conclusion permit me to say that if Dr. McClellan has done the Society any injustice, it was from want of information; seeing the report published in the Report of the Board of Health, he would naturally infer that it belonged to that Board.

While I am ready and willing to grant all the credit due to the Society, I do protest against Dr. Danforth's view of the case; for if ordering the examinations and paying for them is not having some connection with them, then publishing the report in ours, accredited to him, does not give us any connection with it.

Respectfully Yours,

BEN C. MILLER, *Sanitary Sup't.*

